# Cornelia de Lange Syndrome Caused by an Intragenic Heterozygous Deletion in *RAD21* Detected through Very-High-Resolution Chromosomal Microarray Analysis

**DOI:** 10.3390/genes14122212

**Published:** 2023-12-14

**Authors:** Hugo H. Abarca-Barriga, Renzo Punil Luciano, Flor Vásquez Sotomayor

**Affiliations:** 1 Instituto de Investigaciones de Ciencias Biomédicas, Universidad Ricardo Palma, Lima 15039, Peru; fvasquezso@insn.gob.pe; 2 Servicio de Genética & Errores Innatos del Metabolismo, Instituto Nacional de Salud del Niño Breña, Lima 15083, Peru; rpunil@insn.gob.pe

**Keywords:** *RAD21*, de Lange syndrome, microarray analysis, gene deletion

## Abstract

Cornelia de Lange syndrome is a genetic and clinically heterogeneous entity, caused by at least five genes. It is characterized by short stature, gestalt facies, microcephaly, neurodevelopmental disorders, and other anomalies. In this report, we present a 13-year-old female patient with microcephaly, cleft palate, polydactyly, short stature, triangular facies, frontal bossing, a bulbous nose, an overfolded helix, limited pronosupination, and an anomalous uterus. No neurodevelopmental disorders were reported. A chromosomal microarray analysis of 6.5 million markers was performed in the proband and her parents. The results showed a de novo heterozygous microdeletion of exons 9–14 within *RAD21*, which confirmed the diagnosis of Cornelia de Lange syndrome type 4. Our patient did not show any neurologic phenotype (until the time of diagnosis), although neurodevelopmental disorders are frequently present in patients with Cornelia de Lange syndrome type 4, and despite carrying a deletion that was larger than previously reported. Therefore, unknown genetic modifiers or intrinsic mechanisms of *RAD21* variants may exist and should be studied.

## 1. Introduction

Cornelia de Lange syndrome (CdLS, OMIM PS122470) comprises a group of conditions that are diverse in their genetic origin and clinical manifestations [1,2]. The prevalence has been reported to be 1/10,000–100,000, although it is thought to be higher [3,4,5]. Five genes (*NIPBL*, *SMC1A*, *HDAC8*, *RAD21,* and *SMC3*) have been associated with CdLS phenotypes of autosomal dominant or X-linked inheritance [3].

Among other clinical manifestations, patients usually show short stature, thick eyebrows, synophrys, a depressed nasal bridge, a short nose, anteverted nares, a long and smooth philtrum, and a thin and tented upper lip vermilion [2]. The prenatal proportional short stature is characterized by anthropometry at birth below percentile 10, followed by postnatal growth below percentile 5 [4].

There is a consensus on the diagnostic criteria of CdLS with a score greater than 11 on the International Consensus Statement [2], including microcephaly, oligodactyly or adactyly, congenital diaphragmatic hernia, development delay, intellectual disability, small hands, short feet, and hirsutism [2].

CdLS type 4 (OMIM # 614701) patients are extremely rare, and their phenotype is associated with heterozygous variants in *RAD21*, although it is not fully understood how variants in this gene cause the syndrome. The most frequent clinical manifestations are motor developmental delay or intellectual disability, thick arched eyebrows, microcephaly, synophridia, short anteverted nostrils, and clinodactyly of the fifth finger [6]. Missense homozygous variants in *RAD21* were associated with Mungan syndrome (OMIM #611376). In zebrafish, these variants appear to manifest as a loss-of-function effect of the *rad21* gene. This effect showed the partial or complete absence of *runx1* expression in the gut during development. The principal clinical manifestation is a chronic intestinal pseudo-obstruction without gestalt facies like CdLS [7,8].

*RAD21* (Gene ID: 5885, MIM *606462) is located on chromosome 8q24.11, spans 14 exons, and encodes RAD21, a 631-residue protein [9]. This protein is a structural component of the cohesion complex, which holds sister chromatids together until anaphase, ensuring the correct chromosomic segregation during mitosis [9], and is highly conserved in eukaryotes [9,10,11]. Additionally, RAD21 is involved in the DNA repair progression of apoptosis, centrosome cycles, gene expression, and hematopoiesis [12,13,14,15,16,17]. Regarding cohesion complex function, RAD21 is one of its components, working together with SMC1A, SMC3, and SSC3, forming a tripartite ring-like structure; therefore, variants in these genes comprise the etiology of CdL syndrome; see also [11].

In this study, we describe a female patient presenting with short stature, microcephaly, and other congenital malformations, without intellectual disability. High-resolution chromosome microarray analysis (CMA) showed a previously undescribed de novo heterozygous deletion, comprising several exons in *RAD21*. This copy number variation (CNV) is associated with CdLS type 4. We review the previously reported clinical characteristics of CdLS type 4 patients and compare them with those of the patient in the current study.

## 2. Results

### Clinical Evaluation

**Figure 1:**
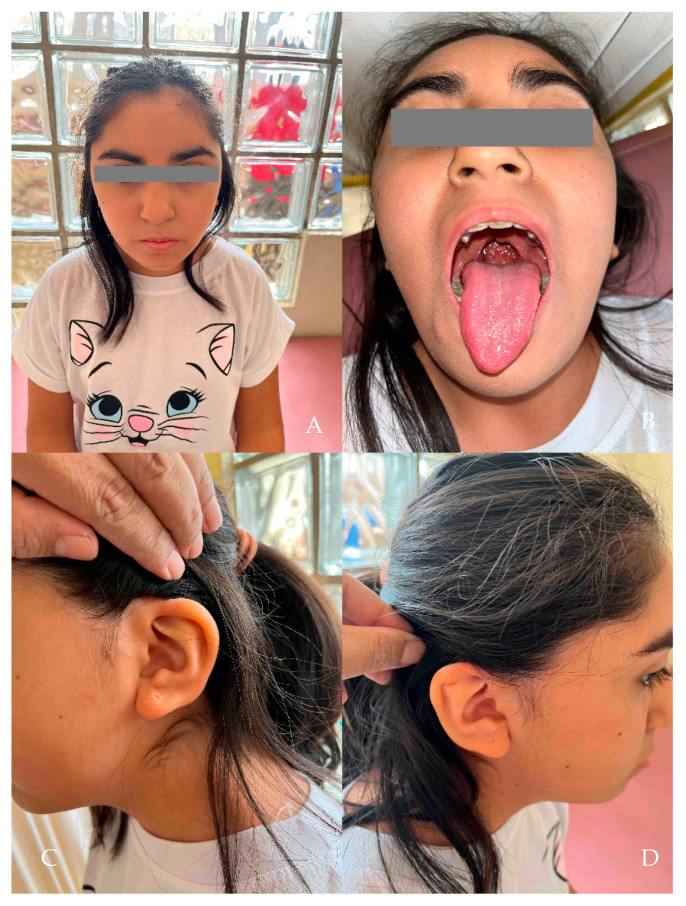
(**A**) The patient with triangular facies and large eyelashes, and without synophrys. (**B**) The palate repaired of the cleft without the uvula; (**C**) and (**D**) the overfolded helix and prominent helix.

**Figure 2:**
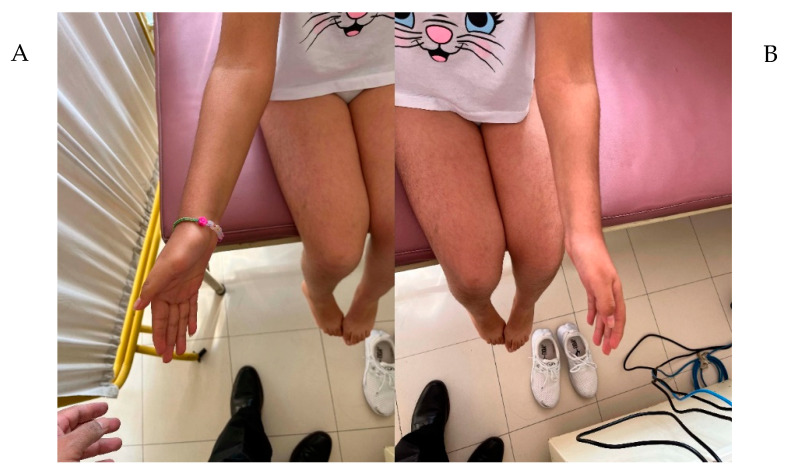
(**A**) and (**B**) limited pronosupination in both arms.

**Figure 3:**
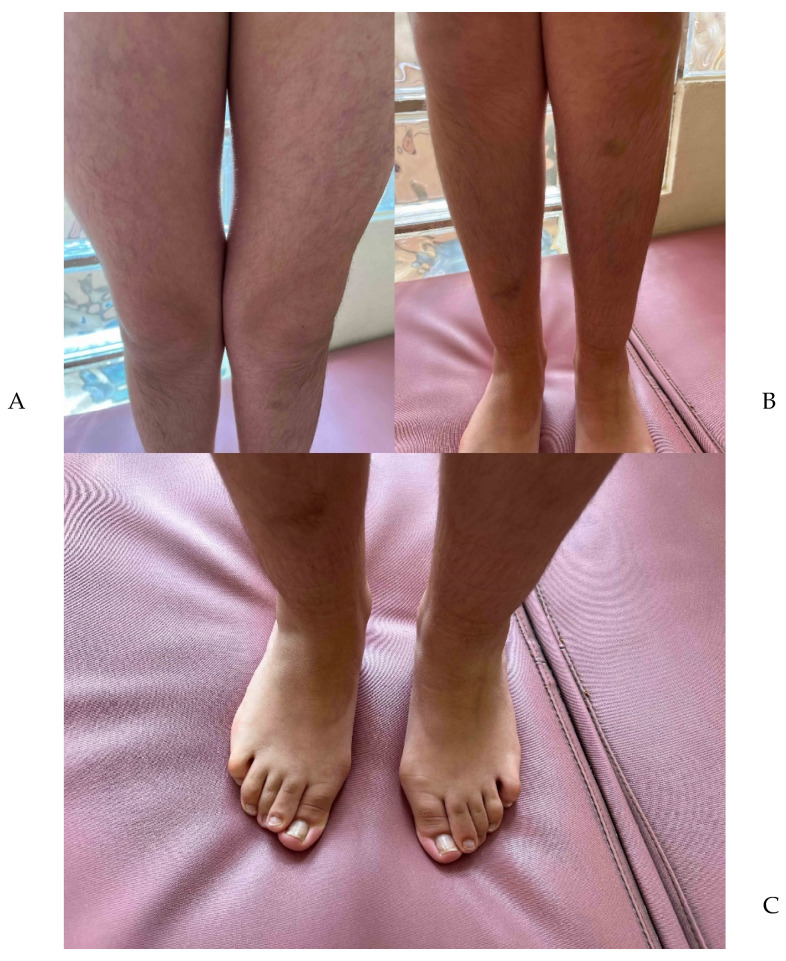
(**A**) The patient with *cutis marmorata* and hypertrichosis. (**B**) The presence of hypertrichosis of the limbs. (**C**) Overlapping toes, *hallux valgus*, and clinodactyly of the fourth and fifth bilateral toes.

The patient, a female aged 13, is the second child of an unrelated couple of Peruvian origin (27-year-old mother and 32-year-old father). The proband was born via cesarean section due to preeclampsia, premature rupture of membranes, and pelvic narrowing. The weight and height at birth were 3190 g (p30) and 52 cm (p86), respectively. Her motor development and school performance were adequate. At twelve days old, her clinical characteristics were a head circumference of 32 cm (−2.695 SD), micrognathia, hypotelorism, a short neck, pre-axial polydactyly, and cleft lip and palate. At 2 years of age, the patient underwent cleft palate repair surgery. At two months, her anthropometry values included a normal height (59 cm, p98) and microcephaly (HC = 35.5 cm, −3.21 SD). At 3 years of age, she presented with short stature (98.4cm, −3.346 SD), triangular facies, a broad and prominent forehead, a bulbous nose, a repaired palate (Figure 1A,B), a bilateral overfolded helix (Figure 1C,D), bilateral prono-supination limitation (Figure 2A,B), cutis marmorata (Figure 3A), hypertrichoses with a predominance in the lower limbs (Figure 3B), and overlapping toes (Figure 3C). At 12 years of age, a pelvic ultrasound showed a bicornuate uterus of 4.3 cm. The IGF-1 was low (167 ng/mL; VR 191–482 ng/mL), with post-stimulation growth to 60 min of 1.85 ng/mL, and to 90 min of 1.81 ng/mL. A full spine X-ray did not show any alterations, while a forearm X-ray showed diaphyseal curvature of the radius. At 13 years of age, a full abdomen MRI was performed, which showed a normal upper abdomen and an arcuate uterus. A TORCH test was negative. 

Clinical diagnoses of some genetic diseases were not suspected, although we did not use any software (e.g., Face2Gene 2.2 ^®^); furthermore, as a first approach, a karyotype test was ordered (due to short stature), and a normal result was obtained (46,XX). Because the patient’s phenotype (the absence of neurodevelopment disorders) was more likely caused by intragenic variants (single or multiple nucleotides) than a copy number variation, a high-resolution CMA test was performed. The result (arr[GRCh38] 8q24.11(116845458_116854956)x1) showed a 9499 bp heterozygous deletion in 8q24.11, comprising six exons (9–14) within *RAD21*. The carrier status of the parents was determined via performance of the same high-resolution CMA, which showed no deletion present in the parents. Subsequently, whole-exome sequencing was performed without pathogenic or likely-pathogenic single-variant nucleotides.

## 3. Materials and Methods

### 3.1. Ethical Statement

Following the Instituto Nacional de Salud del Niño regulations, informed written consent was obtained from the patient’s parents.

### 3.2. Chromosome Microarray Analysis

Genomic DNA was isolated from whole blood using the gSYNC™ DNA Extraction Kit (Geneaid Biotech Ltd., New Taipei City, Taiwan). Total genomic DNA (100 ng) was amplified, labeled, and hybridized using CytoScan™ XON array protocols (Thermo Fischer Scientific, Waltham, MA, USA), according to the manufacturer’s instructions. The array specifications included 6,550,000 nonpolymorphic markers and approximately 300,000 SNP markers. CEL files obtained via scanning the arrays were analyzed using Chromosome Analysis Suite (ChAS) software v4.3 (Affymetrix) and Genome Build GRCh38 (hg38). Gains that affected a minimum of 50 markers, losses that affected a minimum of 25 markers, and loss of heterozygosity (LOH) regions that expanded over 5 Mb were initially considered.

## 4. Discussion

Chromatinopathies are caused by variants in the proteins responsible for chromatin remodeling and transcriptional regulation. These variants cause a global deregulation of gene expression and, consequently, favor the appearance of intellectual disability, delayed psychomotor development, and behavioral disorders [3]. CdLS is a chromatinopathy and shares the characteristics previously described with Rubinstein–Taybi, Coffin–Siris, Wiedemann–Steiner, and KBG syndromes [3].

CdLS is caused by genetic variants in the ring-like cohesin protein complex genes. The complex comprises two regulators (NIPBL and HDAC8) and four structural proteins (SMC1A, SMC3, SCC3 and RAD21). Variants in *NIPBL* have been found in approximately 70% of patients. Variants in other subunits or regulators of the complex are also responsible for CdLS [3]. The cohesin complex maintains chromatin structure during cell division and transcription regulation [18]. RAD21 binds to the SMC1-SMC3 heterodimer by its conserved carboxyl-terminal and amino-terminal protein domain, respectively. RAD21 also binds SCC3 (SA1/SA2) by its STAG domain [19].

Most variants found in *RAD21* are single-nucleotide variants (SNVs). However, several patients diagnosed with CdLS have been shown to carry CNVs, which span several genes, including *RAD21* [8]. Here, we show a previously undescribed variant, an intragenic de novo microdeletion which comprises exons 9–14 within *RAD21*, in a patient with no pre-established genetic diagnosis. The deletion of these exons generates a truncated protein that lacks the STAG domain and the C-terminal region, both necessary for the interaction with SMC1 and the stabilization of the cohesin complex [8]. This gene is haploinsufficient (ClinGen ^®^ shows LOEUF = 0.26, pLI score = 1, index haploinsufficiency = 4.51, and HI score = 3), and this deletion has a dominance effect.

To date, including the patient described in the present study, 27 patients with *RAD21* variants have been reported. In many of these studies, only the genetic variants have been described, without the clinical characteristics (Table 1) [6,8,20,21,22,23,24,25,26,27,28,29]. It can also be observed that the distribution of CdLS has been similar among males and females, with the time of diagnosis having a median of 5.5 years.

The most-described clinical manifestations were microcephaly, broad eyebrows, synophrydia, long eyelashes, a long philtrum, clinodactyly, and psychomotor developmental delay (Table 1). From the 27 CdLS cases, 7 were caused by CNVs (deletions including *RAD21)*, 7 were frameshift variants, 7 were nonsense variants, and the rest were missense and splicing variants [6,8,20,21,22,23,24,25,26,27,28,29]. In one of the reported cases, even though different technologies were used, such as Face2Gene and the CdLS diagnosis score [28], the variant described is predicted as “probably benign” according to Varsome and as “uncertain” according to Franklin [31,32]. Post hoc analysis of the phenotype of our patient did not show clinical criteria of CdLS.

It is important to highlight that the current commonly used genetic platforms are incapable of detecting all genetic variants. Moreover, a lack of information and evidence hinders the classification of most of the variants found in these studies. Until the technology and analyses are improved, we consider that CMA (normal- and high-resolution) and next-generation sequencing (NGS) should be used complementarily to synergistically improve their power of variant detection. Going forward, third-generation sequencing could replace conventional methods because it can detect almost all types of variants including SNVs and CNVs, while NGS primarily detects SNVs and CNVs, failing to achieve the same level of sensitivity as CMA [30].

This study is the first to show a de novo intragenic deletion in *RAD21*, detected via high-resolution CMA, that is associated with CdLS type 4. In addition, the patient described in this study showed clinical manifestations previously unreported for CdLS type 4 patients, such as uterus malformations and preaxial polydactylies [6,8,20,21,22,23,24,25,26,27,28,29]. These characteristics could be unusual signs of CdLS or could be the result of other variants, such as SNVs, that cannot be detected via the techniques used in this study. To test this hypothesis, an NGS analysis is suggested. However, considering the clinical manifestations, the high-resolution CMA results obtained for the proband and her parents, and the literature reviewed, we believe that the CNV found in the present study is likely pathogenic and could cause the patient’s phenotype.

## Figures and Tables

**Table 1:** Clinical characteristics of patients with Cornelia de Lange syndrome type 4.

	Deardorff et al. 2012 [19] (n = 6)						Minor et al. 2014 [21] (n = 2)		Ansari et al. 2014 [20] (n = 1)	Lee et al. 2014 [22] (n = 1)	Martínez et al. 2017 [23] (n = 1)	Boyle et al. 2017 [24] (n = 4)				Yuan et al. 2019 [25] (n = 2)		Kruszka et al. 2019 [26] (n = 3)			Gudmunson et al. 2019 [6] (n = 1)	Dorval et al. 2019 [27] (n = 1)	Latorre-Pellicer et al. 2020 [28] (n = 3)			Lei et al. 2020 [29] (n = 1)	Abarca et al. 2023 [30] (n = 1)	Total (N = 26)	
	1	2	3	4	5	6	1	2				1	2	3	4	1	2	1	2	3			1	2	3				(%)
Sex	*	M	*	*	*	*	M	M	F	*	*	F	F	F	F	*	*	F	M	M	M	M	F	F	M	M	F	F = 9/M = 9/* = 9	
Age (years)	*	*	*	*	*	*	3	12		4	*	26	*	*	*	*	*	7	14	2	1.25	5	3	5	8	6	13	Mdn = 5.5	
Congenital microcephaly	*	+	*	*	*	*	*	*	*	*	*	*	*	*	*	*	*	*	*	*	*	−	*	*	*	*	+	2/3	66.7
Small for gestationatl age	*	+	*	*	*	*	+	+	*	*	*	*	*	*	*	*	*	*	*	*	−	−	*	*	*	*	−	3/6	50
Low weight	+	+	−	*	−	*	+	−	−	*	*	*	*	*	*	*	*	*	*	*	+	*	*	*	*	+	+	4/10	60
Short stature	+	+	−	+	−	*	+	−	−	*	*	*	*	*	*	*	*	−	−	−	−	*	*	*	*	+	+	6/14	42.9
Holoprosencephaly	*	*	*	*	*	*	*	*	*	*	*	*	*	*	*	*	*	+	+	−	*	−	*	*	*	*	−	2/5	40
Microcephaly	+	+	+	+	+	+	+	+	+	+	*	+	+	*	*	*	*	*	+	+	+	+	*	*	*	*	+	17/17	100
Prominent forehead	−	*	*	*	−	+	+	−	*	*	*	−	*	*	*	*	*	*	*	*	*	*	*	*	*	*	+	3/7	42.9
Braquicephaly	+	−	*	*	*	*	−	+	*	*	*	*	*	*	*	*	*	*	*	+	*	*	*	*	*	*	−	3/6	50
Thick eyebrow	+	+	+	+	+	+	+	+	+	+	*	+	*	*	*	*	*	*	+	+	+	+	*	*	*	+	+	17/17	100
Synophrys	+	−	+	+	+	+	+	+	*	*	*	*	*	*	*	*	*	+	*	+	−	+	*	*	*	*	−	10/13	76.9
Nistagmus	−	*	*	*	*	*	+	−	*	*	*	*	*	*	*	*	*	*	*	*	*	*	*	*	*	*	−	1/4	25
Hyperopia	−	*	*	*	*	*	−	+	*	*	*	*	*	*	*	*	*	*	*	*	*	*	*	*	*	*	−	1/4	25
Long eyelashes	+	+	+	+	+	+	−	+	*	+	*	*	*	*	*	*	*	*	*	*	+	+	*	*	*	+	+	11/14	92.3
Long philtrum	−	+	+	+	−	+	−	+	*	*	*	+	*	*	*	*	*	*	+	+	+	+	*	*	*	*	+	11/14	78.6
Strabismus	*	*	*	*	*	+	+	−	*	*	*	*	*	*	*	*	*	*	*	*	*	*	*	*	*	*	−	2/4	50
Ptosis	+	*	*	*	+	+	+	−	*	*	*	+	*	*	*	*	*	*	*	*	+	*	*	*	*	*	+	7/8	87.5
Micrognathia	+	−	+	+	−	−	+	−	*	*	*	−	*	*	*	*	*	*	*	*	+	*	*	*	*	+	+	7/12	58.3
Cleft palate	+	−	*	*	+	*				*	*	*	*	*	+	*	*	+	*	+	+	*	*	*	*	+	+	8/9	88.9
Broad uvula	*	*	*	*	*	*	−	+	*	*	*	*	*	*	*	*	*	*	*	*	*	*	*	*	*	*	−	1/3	33.3
Short nose	+	*	*	*	*	−	+	−	*	*	*	+	*	*	*	*	*	*	*	+	+	*	*	*	*	*	+	6/8	75
Nares anteverted	*	*	*	*	*	+	−	+	*	*	*	+	*	*	*	*	*	*	+	*	+	*	*	*	*	+	−	6/8	75
Depressed nasal bridge	+	+	*	*	*	−	+	−	*	*	*	+	*	*	*	*	*	*	*	*	*	*	*	*	*	+	−	5/8	62.5
High nasal bridge	*	*	*	*	*	+	−	+	*	*	*	−	*	*	*	*	*	*	*	*	*	*	*	*	*	*	+	3/5	60
Increased posterior angulation of ear	+	*	*	*	*	+	+	−	*	*	*	−	*	*	*	*	*	*	*	*	*	*	*	*	*	*	+	4/6	66.7
Low-set ear	*	*	*	*	*	*	−	+	*	*	*	−	*	*	*	*	*	*	+	+	*	*	*	*	*	*	−	3/6	50
Deafness	−	*	*	*	*	*	−	−	*	*	*	*	*	+	+	*	*	*	*	*	−	*	*	*	*	*	−	2/7	28.6
Hirsutism	−	−	*	+	*	−	−	+	*	*	*	*	*	*	*	*	*	*	*	*	*	*	*	*	*	*	+	3/7	42.9
Clinodactyly of fitth finger	+	*	*	−	*	+	+	+	*	*	*	*	*	*	*	*	*	*	+	+	+	+	*	*	*	+	−	9/11	81.8
Syndactily of 2-3 fingers	*	*	*	−	*	+	−	+	*	*	*	*	*	*	*	*	*	*	*	*	−	*	*	*	*	*	−	2/6	33.3
Overlapping toes (2-3)	*	*	*	−	*	*	+	−	*	*	*	*	*	*	*	*	*	*	*	*	*	*	*	*	*	*	+	2/4	50
Syndactily of 2-3 toes	*	*	*	−	+	+	+	−	*	*	*	*	*	*	*	*	*	*	*	*	*	*	*	*	*	*	−	3/6	50
Single transverse palmar crease	*	*	*	*	*	*	+	−	*	*	*	*	*	*	*	*	*	*	*	*	*	*	*	*	*	*	−	1/3	33.3
Radio-ulna synostosis, limited elbow range of motion	+	−	*	−	+	*				*	*	*	*	+	+	*	*	*	*	*	−	*	*	*	*	*	+	5/8	62.5
Hemivertebra/butterfly vertebra	+	+	*	*	+	*				*	*	*	*	*	*	*	*	*	−	−	*	*	*	*	*	+	−	4/10	57.1
Pectus excavatum	*	*	*	*	−	*	−	+	*	*	*	*	*	*	*	*	*	*	*	*	*	*	*	*	*	*	−	1/4	25
Pectus carinatum	*	*	*	*	+	*	−	−	−	*	*	*	*	*	*	*	*	*	*	*	*	*	*	*	*	*	−	1/5	20
Congenital heart disease	*	*	*	*	+	*				*	*	*	*	*	+	*	*	−	*	−	*	*	*	*	*	+	−	3/6	50
Gastroesophageal reflux disease	+	*	*	*	+	*	−	+	*	*	*	*	*	*	*	*	*	*	+	+	+	+	*	*	*	*	−	7/9	77.8
Hypospadias	*	*	*	*	*	*	+	−	n/a	*	*	n/a	n/a	n/a	n/a	*	*	n/a	*	*	*	*	n/a	n/a	*	*	n/a	1/2	50
Cryptorchidism	*	*	*	*	*	*	+	−	n/a	*	*	n/a	n/a	n/a	n/a	*	*	n/a	*	*	+	*	n/a	n/a	*	*	n/a	2/3	66.7
Bilateral inguinal hernia	*	*	*	*	*	*	+	−	n/a	*	*	n/a	n/a	n/a	n/a	*	*	n/a	*	*	*	*	n/a	n/a	*	+	n/a	2/3	66.7
Congenital diaphragmatic hernia	*	*	*	*	*	*	*	*	n/a	*	*	n/a	n/a	n/a	n/a	*	*	n/a	*	*	+	*	n/a	n/a	*	*	n/a	1/1	100
Bifid scrotum	*	*	*	*	*	*	+	−	n/a	*	*	n/a	n/a	n/a	n/a	*	*	n/a	*	*	*	*	n/a	n/a	*	*	n/a	1/2	50
Penoscrotal transposition	*	+	*	*	*	*	+	−	n/a	*	*	n/a	n/a	n/a	n/a	*	*	n/a	*	*	*	*	n/a	n/a	*	*	n/a	2/3	66.7
Development delay	−	*	+	*	+	*	+	+	*	*	*	*	*	*	*	*	*	+	+	+	+	−	*	*	*	*	−	8/11	72.7
Language delay	−	+	+	*	+	*	+	−	*	+	*	*	*	*	*	*	*	*	*	*	*	+	*	*	*	*	−	6/9	66.7
Motor delay	−	+	*	*	+	*	+	−	*	*	*	*	*	*	*	*	*	*	*	*	*	−	*	*	*	*	−	3/7	42.9
Autistic spectrum disorders	−	*	*	*	+	*	+	.	*	*	*	*	*	*	*	*	*	*	*	*	*	*	*	*	*	*	−	2/4	50
Specific learning disorders	−	*	*	*	*	*	−	+	*	*	*	+	+	+	+	*	*	*	*	*	*	*	*	*	*	*	−	5/8	62.5
Intellectual disability	−	*	+	+	*	*				*	*	*	*	*	*	*	*	*	*	*	−	−	*	*	*	+	−	3/7	42.9
ADHD	−	*	*	*	*	*	+	+	*	*	*	*	*	*	*	*	*	*	*	*	*	*	*	*	*	*	−	2/4	50
Cutis marmorata	+	+	*	−	*	−	*	*	*	*	*	*	*	*	*	*	*	*	*	*	*	*	*	*	*	*	+	3/5	60
Preaxial polydactyly																											+	1/1	100
Bicornuate uterus																											+	1/1	100
Clinical scores	*	*	*	*	*	*	*	*	*	*	*	*	*	*	*	*	*	*	*	*	*	*	*	−	10	*	*		
Variant	8:117,708,713–121,024,193 ❡	8:117,640,909–119,330,085 ❡	8:117237890–122631628 ❡	8:116,950,003–118,944,486 ❡	c.1127C>G	c.1753T>C	Deletion exon 13	c.592_593dup	*	c.1808T>C	c.86G>A	c.704delG	c.704delG	c.704delG	c.704delG	c.1550dupC	c.1161+1G>A	c.1548delinsTC	c.589C>T	c.1217_1224del	c.1774_1776del	c.943_946del	c.1382C>T	8:117765326_122494596 ❡	8:117765326_118270323 ❡	8:115443000_123744000₣	8:116845458_116854956₣		
Exon	1–14	1–14	1–14	1–14	9	14	13	3		14	2	7	7	7	7	12	Intron 10	12	6	10	14	9	11	1–14	1–14	1–14	9–14		
							665 pb		*																				
Type of variant	CNV	CNV	CNV	CNV	*Missense*	*Missense*	*Frameshift*	*Frameshift*	*Splicing site*	*Missense*	*Codon stop*	*Codon stop*	*Codon stop*	*Codon stop*	*Codon stop*	*Frameshift*	*Splicing site*	*Frameshift*	*Codon stop*	*Frameshift*	*Frameshift*	*Codon stop*	*Missense*	*CNV*	*CNV*	*CNV*	Frameshift		
Classification										LP	LP	P	P	P	P			P	P	P	LP	P	VUS−LB	P	P	P	P		

n/a = not applicable; VUS-LB = variant of uncertain significance or likely benign; * non-descript. F = female; M = male; Mdn = median; P = pathogenic; LP = likely-pathogenic; ADHD = attention deficit and hyperactivity disorder; ❡ = hg18; ₣ = hg19.

## Data Availability

All data underlying the results are available as part of the article and no additional source data are required.
